# Case Report: Blood single-cell analysis of a IVB high-grade serous ovarian cancer patient presenting a favorable prognosis

**DOI:** 10.3389/fonc.2025.1697863

**Published:** 2025-11-24

**Authors:** Martyna Wolańska, Michał Sieczczyński, Krzysztof Pastuszak, Anna Samelak-Czajka, Paulina Jackowiak, Natalia Bednarz-Knoll, Sylwia Łapińska-Szumczyk, Dagmara Klasa-Mazurkiewicz, Anna J. Żaczek, Anna Supernat

**Affiliations:** 1 Laboratory of Translational Oncology, Intercollegiate Faculty of Biotechnology of the University of Gdańsk, The Medical University of Gdańsk, Gdańsk, Poland; 2 Department of Software Engineering, Faculty of Electronics, Telecommunications and Informatics, Gdańsk University of Technology, Gdańsk, Poland; 3 Centre of Biostatistics and Bioinformatics, Medical University of Gdańsk, Gdańsk, Poland; 4 Department of Algorithms and Systems Modelling, Faculty of Electronics, Telecommunications and Informatics, Gdańsk University of Technology, Gdańsk, Poland; 5 Laboratory of Single Cell Analyses, Institute of Bioorganic Chemistry, Polish Academy of Sciences, Poznań, Poland; 6 Department of Gynecology, Obstetrics, Gynecologic Oncology, and Gynecologic Endocrinology, University Clinical Center, The Medical University of Gdańsk, Gdańsk, Poland

**Keywords:** HGSOC, liquid biopsy, single cell sequencing, platelet RNA, case report

## Abstract

**Background:**

High-grade serous ovarian cancer (HGSOC) diagnosed at stage IVB typically carries a poor prognosis. Here, we describe a rare case of with an exceptional and sustained response to therapy. To explore potential drivers of this favorable outcome, we combined clinical evaluation with molecular profiling of liquid biopsy samples.

**Methods:**

We employed a multi-platform liquid biopsy approach in peripheral blood samples collected preoperatively. Bulk RNA sequencing was performed on platelet RNA, while single-cell RNA sequencing (scRNA-seq) profiled peripheral blood mononuclear cells (PBMCs). Additionally, circulating tumor cells were identified using imaging flow cytometry (imFC).

**Results:**

Single-cell transcriptomic analysis identified two candidate CTCs (Circulating Tumor Cells): one with an epithelial phenotype and another with a hybrid epithelial–mesenchymal (EMT) phenotype. The EMT CTC showed upregulation of IL12A, genes involved in the mTOR pathway (RPTOR, RICTOR, MTOR), and DNA repair, while the epithelial CTC expressed high levels of VEGFA. ImFC-based assay identified one putative mesenchymal-like CTC. Platelet RNA analysis revealed downregulation of ribosomal genes and upregulation of genes related to cytoskeletal remodeling and adhesion. ANGPT1 was downregulated, while AKT1 were upregulated, putatively indicating mTOR pathway activation. Glycolytic enzymes PKM and PGK1 were strongly upregulated, and reduced expression of DDIT4, HIF1A, CSF2RA, and CSF3R suggested altered stress and cytokine signaling.

**Conclusion:**

This integrative molecular and phenotypic profiling of blood-derived components identified potentially distinct molecular signatures, such as overexpression of IL12, ANGPT1 downregulation and HIF1A downregulation, in the literature described as linked to the patient’s beneficial prognosis. These findings suggest that advanced liquid biopsy techniques may provide complementary insights into prognostic biomarkers and therapeutic targets in HGSOC.

## 1 Introduction

High-grade serous carcinoma is typically diagnosed at the metastatic stage, with 29% cases presenting as stage IV (1). Frequently associated with *TP53* mutations, HGSOC is more aggressive and generally results in poorer outcomes than other histological types of ovarian cancer (2). Despite advances in treatment, HGSOC continues to pose significant challenges in improving long-term outcomes (3).While the prognosis for advanced-stage HGSOC is generally poor, approximately 15% of patients achieve long-term survival of 10 years or more following standard treatment (4). Several factors contribute to prolonged progression-free survival (PFS) and overall survival (OS). These include good performance status (5), adherence to treatment (6, 7), low tumor mutation burden (8), optimal cytoreductive surgery (9), post-treatment CA-125 normalization (10), BRCA1/2 mutations or homologous recombination deficiency (11, 12), long term sensitivity to platinum-based chemotherapy (13), use of PRPP inhibitors or anti-VEGF therapy (14), no residual disease (15), and stable mental well-being (16).

In this case report we investigated the patient who demonstrated an exceptional response to treatment despite stage IV HGSOC. The aim of this case study was therefore to explore putative features that might have contributed to her unique clinical outcome. In addition to established clinical factors, we preoperatively collected a peripheral blood sample, analyzed platelet RNA and profiled blood cells with single-cell RNA sequencing and imaging flow cytometry. This multi-layered liquid biopsy approach provided a level of molecular and cellular resolution that has only recently become feasible. While liquid biopsy techniques, such as circulating tumor DNA (ctDNA) and circulating tumor cells (CTCs), have been studied for early detection, recurrence monitoring, and prognosis in ovarian cancer (17, 18) the integration of platelet bulk RNA sequencing and PBMC scRNA-seq remains largely underexplored, particularly for identifying prognostic immune-metabolic signatures in exceptional responders. To place these findings in context, the patient’s cellular profiles were compared with those from HGSOC patients with poor prognoses, with the aim of identifying transcriptomic differences associated with clinical outcomes.

## 2 Materials and methods

### 2.1 Case description

A 63-year-old woman presented in December 2022 with abdominal discomfort. Standard clinical imaging, diagnostic tests and laparoscopic debulking surgery revealed FIGO stage IVB ovarian cancer with widespread peritoneal, visceral, and supradiaphragmatic lymph node metastases, and a CA-125 level of 2,207 U/mL. Definitive diagnosis was established during laparoscopic debulking surgery, where resected tumor tissue confirmed FIGO stage IVB high-grade serous ovarian cancer. No separate laparoscopic biopsy or pre-operative IHC was performed. The therapy schedule consisted of three neoadjuvant cycles of paclitaxel and carboplatin from February to April 2023, followed by debulking surgery in June 2023, then adjuvant cycles of paclitaxel and carboplatin starting in July 2023 with a temporary pause due to thrombocytopenia and neutropenia in September 2023, and continuation with a series of 18 cycles of bevacizumab from October 2023 through August 2024. Despite the high disease burden, the patient tolerated chemotherapy well, achieved complete microscopic resection (R0), and entered sustained biochemical remission (CA-125–13 U/ml); as of May 2025, she remains alive with no biochemical and radiologic evidence of disease progression.

### 2.2 Data and samples

In this study, we analyzed fractionated preoperative blood samples from a patient with stage IVB high-grade serous ovarian carcinoma (HGSOC), i.e. platelets and peripheral blood mononuclear cells (PBMCs). To better interpret the findings, we compared them with samples from other ovarian cancer patients. These included platelet RNA profiles from 37 ovarian cancer cases, single-cell blood transcriptomes from 13 patients with advanced HGSOC and 3 patients with benign ovarian conditions (genital cord tumor, Brenner tumor, and endometriosis), and blood samples from 23 HGSOC patients tested for circulating tumor cell (CTC) protein markers.

All participants were recruited from the Clinic of Obstetrics and Gynecology, Gynecologic Oncology, and Gynecologic Endocrinology at the Medical University of Gdańsk. Inclusion criteria for patients recruitment were: confirmed HGSOC at stage III or IV, age over 18, and peripheral blood collection prior to any treatment. Control samples were obtained from patients with benign gynecologic conditions. The study was approved by the Independent Ethics Committee of the Medical University of Gdańsk (NKBBN/434/2017), and all participants provided written informed consent, following the Declaration of Helsinki, 1983.

Each peripheral blood sample was collected to an EDTA tube, centrifuged and divided into PBMCs and platelet rich plasma fraction. Putative CTCs were detected, enumerated and phenotyped in PBMCs fractions using Amnis^®^ ImageStreamX MkII (Cytek Biosciences) (19) for imaging flow cytometry with already established immunofluorescent staining. In brief, the antibody panel included antibodies targeting pan-keratins (pan-K; epithelial marker), vimentin (V; mesenchymal marker), alpha-smooth muscle actin (α-SMA; myofibroblast/mesenchymal marker), CD29 (integrin β1; adhesion marker), CD31 (PECAM-1; endothelial marker), and CD45 (leukocyte common antigen; hematopoietic marker), as previously described (19, 20). In addition, nuclei were counterstained with 4′,6-diamidino-2-phenylindole (DAPI) to enable DNA content, while side scatter (SSC) was used to capture information on intracellular granularity and structural complexity (19, 20). Platelets were bulk RNA-sequenced according to thromboSeq protocol (21). PBMCs, together with co-isolated single platelets, were isolated and collected using density gradient centrifugation over Histopaque (Sigma-Aldrich, USA). Approximately 20,000 cells per sample were combined with a master mix containing reverse transcription reagents and loaded onto the Chromium Next GEM Chip G. The Chromium Next GEM Single Cell 3′ Reagent Kit (10x Genomics, USA) was used according to the manufacturer’s protocol to generate single-cell transcriptomics libraries. The resulting single cell RNASeq libraries were sequenced on an Illumina NovaSeqX instrument with a paired-end 150-bp approach aiming for 10–000 cells/samples, with 400 mln total reads per one library.

### 2.3 Bioinformatic processing and quality check

The reads were demultiplexed with bcl2fastq2-v2.20.0 and converted to FASTQ format using CellRanger mkfastq. Unique Molecular Identifier (UMI) count matrices representing raw gene expression data were produced with the standard Cell Ranger software (v5.01) from 10x Genomics, employing the GRCh38 human reference genome for mapping. This processing involved filtering out low-quality cells and potential doublets, as well as removing apoptotic cells. Specifically, cells with fewer than 2000 reads overall, cells expressing fewer than 500 unique genes, and cells with over 10% mitochondrial RNA were excluded from further analysis. Following quality control, each read count matrix was normalized to the total expression, scaled by a factor of 10,000, and log-transformed. Quality check as well as normalization was done in R (version 4.4.1) Seurat library (version 5.2.1). Pseudobulk of specific cell types were based on raw counts and normalized using simple CPM (counts per million) method.

Cell type identification on log-normalized and scaled data was performed using the UCell algorithm, a rank-based gene set scoring method implemented in the UCell R library (version 2.8.0). For each cell type, a set of marker genes was curated through a comprehensive literature review and PanglaoDB (22). Cells were subsequently assigned to the cell type with the highest UCell score. This approach facilitated robust cell type annotation across the dataset. Detection of CTC candidates in single cell sequencing dataset was also performed. Sets of marker genes associated with cell types and CTC candidates are provided as **Supplementary Table S1**.

For bulk RNA-seq analysis, raw RNA-seq reads in FASTQ format were processed using a workflow adapted from the thromboSeq protocol (20). First, adapters were trimmed with Trimmomatic (v0.22) and the resulting reads were aligned to the human reference genome (hg38) using STAR (v.2.7.9a). Reads were then assigned to genes with HTSeq (v.0.6.1), guided by the Ensembl GRCh38–104 annotation. The resulting count data were normalized using a simple CPM method.

## 3 Results

### 3.1 Clinical presentation

**Figure 1:**
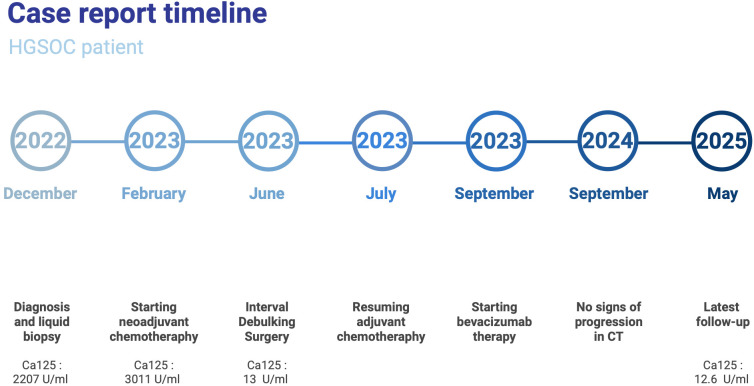
Timeline summarizing key clinical events in the patient’s care.

A 63-year-old female presented in December 2022 with abdominal symptoms. Her age was close to the mean for ovarian cancer patients (mean ~62 years, SD ~15), she maintained a good performance status. Although not young - an age factor sometimes linked to worse prognosis - her response to treatment was remarkably favorable resulting in over 33 months of survival and still alive at the time of the manuscript submission Despite the advanced stage of the disease, her general condition allowed for full adherence to treatment. **Figure 1** Showcases a timeline with relevant data from the episode of care.

At diagnosis, the patient had a high metastatic burden, with extensive disease involving both sides of the diaphragm, visceral organs, and lymph nodes, classifying her as FIGO IVB. Metastases included supradiaphragmatic lymph node involvement, a single thoracic lesion, numerous infiltrations in the colon, peritoneal implants in the pelvis, bilateral diaphragmatic seeding, and dissemination in both organs and lymph nodes. Despite the significant metastatic spread, she continues to do well (as of 19th May 2025). At diagnosis, CA-125 levels were significantly elevated at 2,207 U/mL. Following neoadjuvant chemotherapy, the highest recorded CA-125 level was 3,011 U/mL (13th February 2023). However, post-treatment, a steady decline was observed: 17 U/mL on 24 April 2023, 13 U/mL on 19 June 2023, and 12.6 U/mL at the most recent follow-up, indicating biochemical remission.

On 19th June 2023, the patient underwent cytoreductive surgery achieving complete tumor resection (R0). Procedures included hysterectomy, upper rectum and sigmoid colon resection, omentectomy, peritonectomy, resection of the round ligament of the liver, and HIPEC (cisplatin). Pathological assessment confirmed a chemotherapy response score (CRS) of 3 (23), suggesting an excellent response to systemic therapy. No residual disease was detected postoperatively using standard clinical imaging.

Genetic testing was performed, including the following genes: *BRCA1* and *BRCA2* (core homologous recombination repair factors), PALB2 (stabilizer and partner of BRCA2), CHEK2 (involved in homologous recombination pathway signaling), and TP53 (critical for DNA damage response and cell cycle regulation). A pathogenic BRCA2 mutation, NM_000059.4(BRCA2): c.5993_5997dupAAGTG p.(Phe2000Lysfs6), classified as class I, was identified. Additionally, a likely pathogenic mutation in PALB2, NM_024675.4(PALB2): c.3114-44_3129del p, classified as class II, was also detected. These homologous recombination repair defects are known to confer platinum sensitivity and may explain the patient’s excellent chemotherapy response (CRS 3) and sustained remission. They also support the transcriptomic signals observed in candidate CTCs, suggesting enhanced DNA damage response activity, and indicate potential benefit from PARP inhibition in future treatment lines.

Neoadjuvant chemotherapy with paclitaxel and carboplatin was initiated on 22nd February 2023 and well tolerated, with subsequent adjuvant chemotherapy with paclitaxel carboplatin resumed on 26th July 2023 for four additional cycles. Given the high disease burden at diagnosis and the lack of genetic testing results at the time of decision-making regarding maintenance treatment (information on the presence of a pathogenic BRCA2 variant was received later), bevacizumab was introduced from September 2023 to August 2023 to enhance treatment efficacy.

Adherence to treatment was consistently high. The patient completed all planned chemotherapy cycles and maintenance therapy without dose reductions. A temporary delay in October 2023 due to thrombocytopenia and neutropenia did not affect treatment continuation. Her mental health remained stable throughout treatment, with no reported psychological distress or treatment-related emotional deterioration.

At the latest follow-up (19 May 2025), the patient remained in good general condition, with stable weight (77 kg, BMI 28) and no reported toxicities. Serial CT imaging, most recently in September 2024, demonstrated no disease progression since March 2024. Despite an initial FIGO IVB classification and high tumor burden, she achieved prolonged remission, highlighting the known impact of optimal cytoreductive surgery, platinum sensitivity, BRCA2 mutation status, and anti-VEGF therapy.

### 3.2 Molecular evaluation of CTCs candidates using flow cytometry and single cell RNA sequencing

**Figure 2:**
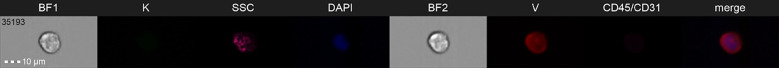
Putative mesenchymal-like circulating tumor cell (CTC) detected in peripheral blood using Amnis^®^ ImageStream^®^X Mk II (40× magnification). The cell was identified by the immunofluorescent panel pan-keratin (K), vimentin (V), DAPI, CD45, and CD31. It shows a DAPI^+^/K^−^/V^+^/CD45^−^/CD31^−^ phenotype, consistent with a putative mesenchymal-like CTC phenotype, and displays an intact, round morphology without attachment to surrounding blood cells. BF1 and BF2, brightfield channels; SSC, side scatter.

The preoperatively collected peripheral blood sample was analyzed using pan-K/V/DAPI/CD45/CD31 immunofluorescent staining and imaging flow cytometry for the presence and phenotype of putative CTCs as described before (22). No “classic”, epithelial-like (K+V-DAPI+CD45-CD31-) or epithelial-mesenchymal (K+V+DAPI+CD45-CD31-) CTCs, but one K-V+DAPI+CD45-CD31- cell was found within 842,799 analyzed DAPI+ cells. This cell identified as putative mesenchymal-like single CTC of ca. the 15 µm diameter revealed an intact and regular circular shape (19), and did not interact with any normal blood cell (**Figure 2**).

When using single cell RNA sequencing data, we identified two CTC candidates among 3123 analyzed cells. Phenotypic analysis revealed that one presumed CTC exhibited an epithelial phenotype, while the other demonstrated an intermediate phenotype between epithelial and mesenchymal (EMT) state. While the epithelial CTC candidate expressed high levels of VEGFA, the EMT CTC candidate showed upregulation of IL12A, genes involved in the mTOR pathway (RPTOR, RICTOR, MTOR), and DNA repair.

### 3.3 Molecular evaluation of PBMCs with single cell RNA sequencing

**Figure 3:**
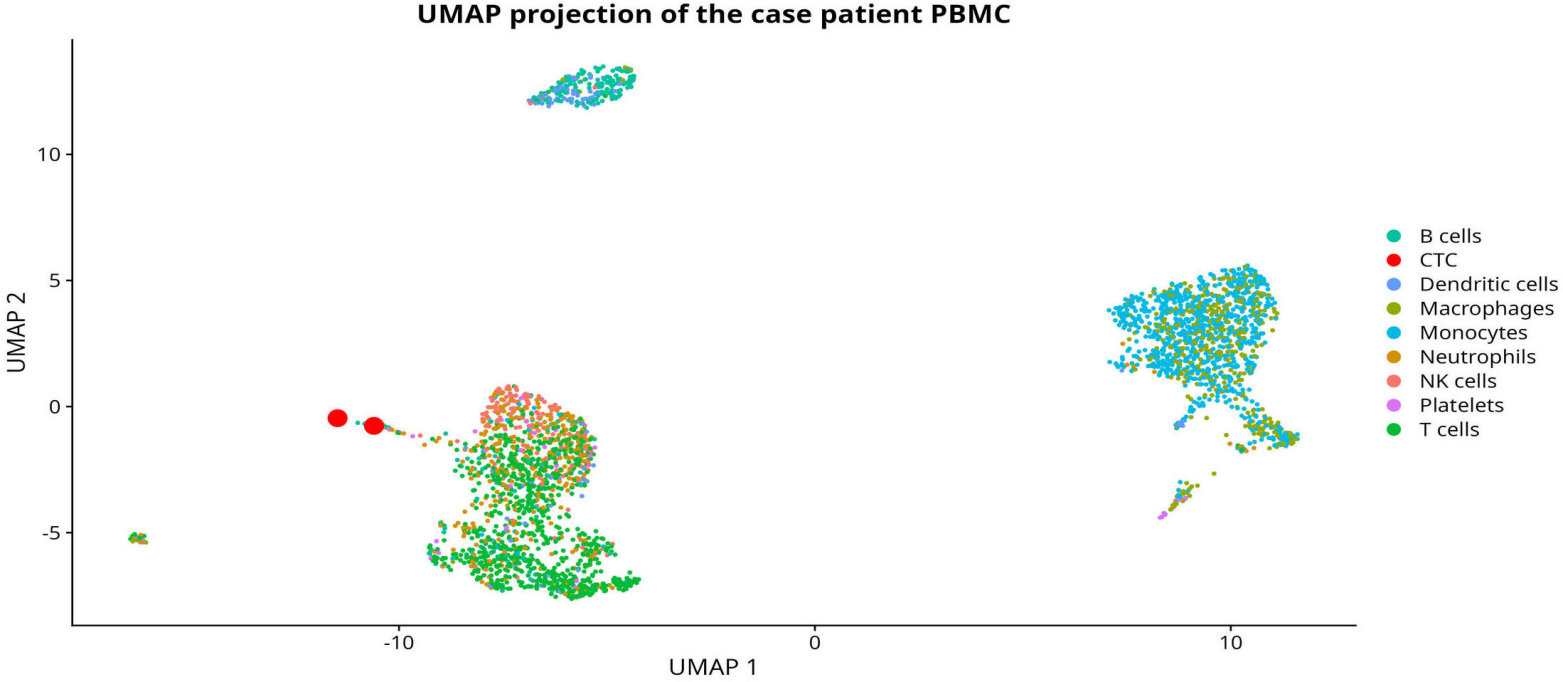
A UMAP projection of the case patient with detected PBMC cell types and highlighted CTCs.

We evaluated pathways related to inflammation, glycolysis, hypoxia, angiogenesis, VEGF and mTOR pathways in B cells, T cells, NK cells and monocytes. We observed strong overexpression of IL12A in all cell types, as opposed to TNF expression, typically high in other patients. Next, we saw very high expression of genes associated with glycolysis: SLC2A1, LDHA, PGK1, PFKFB3, GAPDH, ENO1. Regarding hypoxia, the strongest expression was seen especially for DDIT4, but also for HIF1A, ZEB1, ZEB2. For angiogenesis, we observed downregulation of ANGPT1 and ANGPT2 in T cells, NK cells and monocytes. Given that the mTOR pathway is known to enhance VEGF expression under hypoxic conditions via HIF-1α activation, we also evaluated the expression of these genes. Especially high expression of RICTOR, RHEB, AKT1, PRKAA1 was observed in this patient. The VEGFA was upregulated in all analyzed cell types. We also observed ribosomal genes downregulation among all cell types. **Supplementary Tables S3–S5** show mean expression of selected genes of other ovarian cancer patients compared to the case patient among B-cells, T-cells, NK-cells and monocytes, respectively. **Supplementary Figures S1–S4** presents heatmaps that summarize the aforementioned gene expression. Moreover, **Figure 3** presents UMAP projection of the case patient with marked cell types and CTCs.

### 3.4 Molecular evaluation of platelets’ bulk RNA sequencing

In bulk RNA platelets sequencing, we observed a significant downregulation of ribosomal genes for the analyzed case, suggesting a potential reduction in translational activity. Simultaneously, genes associated with cell adhesion as well as actin cytoskeleton organization and actin filament-based processes were upregulated, indicating cytoskeletal remodeling and altered adhesion dynamics.

**Figure 4:**
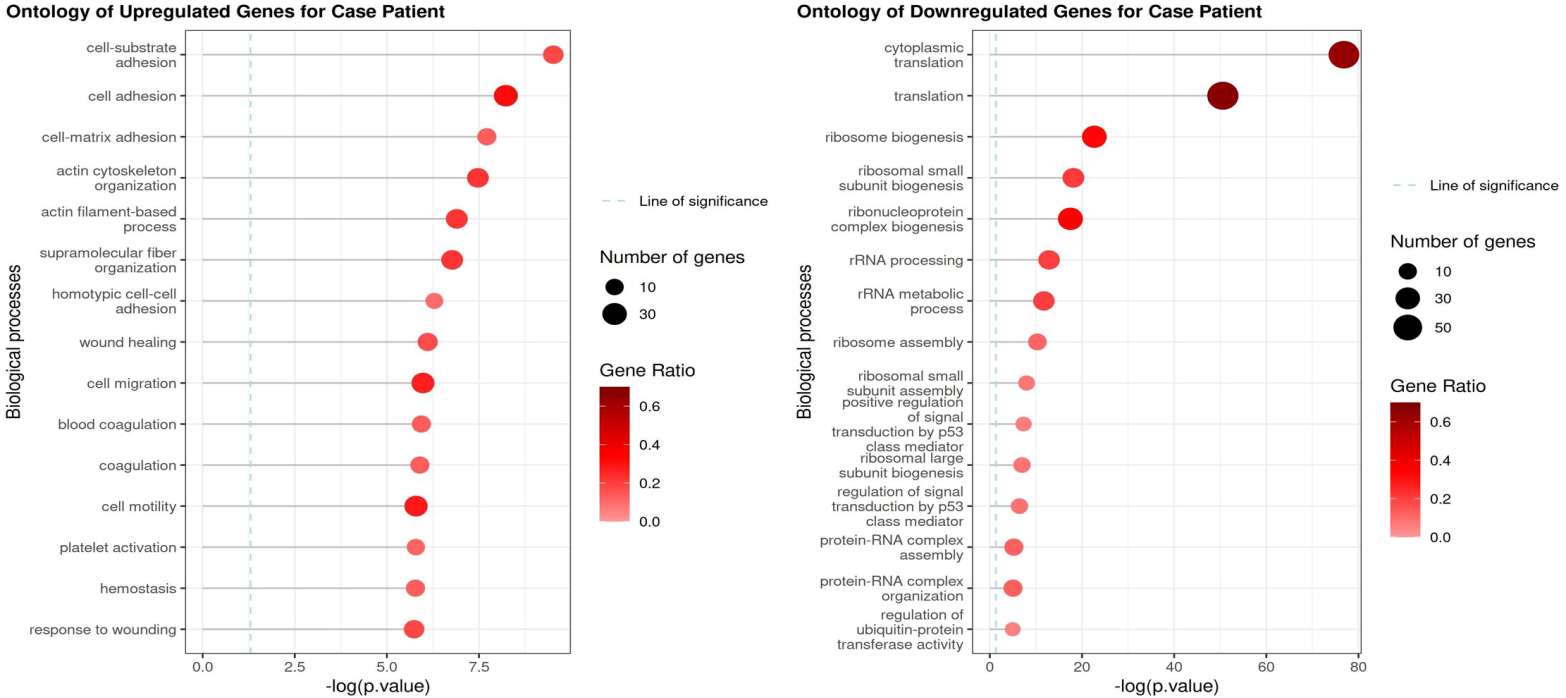
Gene ontology analysis of 100 most significant upregulated and downregulated platelet genes, compared with mean platelet expression of other ovarian cancer patients.

**Figure 4** presents detailed results.

Additionally, ANGPT1 expression was decreased, while AKT1 were upregulated, suggesting activation of mTOR pathway and pathways related to angiogenesis. We also detected a downregulation of DDIT4 and HIF1A, potentially reflecting changes in cellular stress response and hypoxic adaptation. Notably, the glycolytic enzymes PKM and PGK1 were strongly upregulated, pointing to metabolic reprogramming. Lastly, the decreased expression of CSF2RA and CSF3R suggests a potential impairment in cytokine signaling.

## 4 Discussion

This case involves a rare instance of FIGO IVB HGSOC patient with an exceptional clinical outcome spanning over 3 years after treatment, despite extensive metastasis detected at the timepoint of primary diagnosis. Several factors, known from previous studies, might have contributed to this outcome. The patient was diagnosed with pathogenic BRCA2 mutation and PALB2 variant, both related to homologous recombination repair. She achieved complete cytoreduction (R0), presented high sensitivity to platinum-based chemotherapy and received bevacizumab therapy. Given the limitations of a single case report, which inherently restricts generalizability, these elements should be considered potential contributors rather than proven causal factors. A major novelty of this case is the application of single-cell PBMC and platelet RNA profiling, methods increasingly recognized for overcoming the longstanding challenge of CTC detection in ovarian cancer.

Potentially meaningful transcriptomic insights were obtained from just a few individual putative CTCs, validating the feasibility and value of this approach even with limited cellular material (24). Despite the fact that CTCs are difficult to be unquestionably identified as tumor cells, they are frequently associated with invasiveness and treatment resistance (25), and in our analysis an EMT CTC overexpressed DNA repair genes, consistent with platinum sensitivity and BRCA2/PALB2 status (26). This DNA repair gene overexpression in the EMT CTC may represent a compensatory response to the underlying BRCA2 and PALB2 mutations, potentially amplifying the clinical benefit from platinum chemotherapy observed in this patient, though such links remain associative in this case report. Notably, EMT CTC candidate expressed upregulation of mTOR, RICTOR and RPTOR suggesting activation of mTOR signaling and a potential adaptation to hypoxic or anti-angiogenic pressure (27). In contrast, the epithelial CTC displayed VEGFA upregulation, aligning with VEGF pathway activation and the use of bevacizumab in treatment, demonstrating the potential of molecular profiling in optimizing therapeutic strategies and potentially explaining treatment effectiveness. In parallel, this approach was combined with bulk RNA-seq of platelets – a technique that has been previously applied in ovarian cancer primarily for diagnostic purposes, but not in the context of prognosis. Further studies are warranted to explore this area, as platelet transcriptomic profiles may hold potential as prognostic biomarkers in ovarian cancer.

Ovarian cancer is known to shed very few circulating tumor cells into the bloodstream, which constrains the robustness of CTC-based analyses (28, 29). To overcome this limitation and broaden biological insight, we incorporated transcriptomic profiling of PBMCs and tumor-educated platelets, enabling a more comprehensive view of systemic metabolic and immune alterations. Beyond tumor cells, transcriptomic analysis of peripheral blood mononuclear cells and platelets uncovered systemic metabolic and immunologic shifts. To our knowledge, this is the first reported use of such detailed PBMC transcriptomics in ovarian cancer, marking a significant advance in the exploration of tumor-host interactions through liquid biopsy. Specifically, we observed upregulation of glycolysis-related genes (e.g., PKM, PGK1), along with downregulation of ribosomal genes – a pattern consistent with our previous findings (30) and indicative of mTOR pathway activation and altered immune-metabolic states (24). This patient’s tumor metabolism suggests an adaptive survival program, boosting glycolytic flux via PKM and PGK1 to meet energetic and biosynthetic demands, while downregulating ribosomal machinery to conserve resources, potentially in an mTOR-influenced and immune-suppressive context. This typically correlates with aggressive biology, which makes her beneficial prognosis even more remarkable.

A key factor potentially contributing to the favorable prognosis observed in our patient is the elevated expression of interleukin-12 (IL-12) in PBMCs. IL-12 plays a central role in bridging innate and adaptive immunity, promoting the activation and cytotoxic function of NK cells and T cells, and driving a robust anti-tumor immune response. Notably, reduced tumor immunogenicity has been linked to decreased IL-12 production and increased PD-L1 expression, both at the transcript and protein levels, as reported in recent studies on the immunobiology of cold versus hot tumors (31). This suggests that high IL-12 expression may be indicative of an immunologically active, “hot” tumor environment, which is often more responsive to immune surveillance and therapy. Supporting this, a recent preclinical study demonstrated that engineered tumor cells capable of sustained IL-12 secretion led to enhanced anti-tumor immunity and tumor regression *in vivo* (32). These findings align with our observation and reinforce the hypothesis that systemic IL-12 activity, even detectable in peripheral immune cells, could contribute to more effective immune control of tumor progression.

The presence of an EMT-like CTC overexpressing DNA repair genes further suggests that maintenance treatment with PARP inhibitors (e.g., olaparib) may be beneficial in this patient, especially given the known BRCA2 and PALB2 mutations.

Compared to more established liquid biopsy modalities in ovarian cancer, such as ctDNA and CTC enumeration for prognosis and therapy monitoring (17, 18), our method incorporating platelet RNA-seq and PBMC scRNA-seq provides additional insights into immune-metabolic dynamics. Recent studies have shown platelet RNA signatures can predict ovarian cancer prognosis using deep learning models (33), while PBMC scRNA-seq has been used to detect ovarian cancer via T-cell receptor profiling and machine learning (34), but their integration in the context of exceptional responders in HGSOC has not been previously reported.

Next to favorable clinical factors, the distinct transcriptomic profiles observed in PBMCs and platelets highlight the promise of liquid biopsy–derived immune and metabolic markers as potential prognostic tools. Clinically, in this case, the observed molecular signatures - such as mTOR pathway activation and BRCA2/PALB2 mutations - suggest that liquid biopsy approaches might offer insights into potential prognostic markers and therapy response, possibly informing consideration of targeted agents like mTOR or PARP inhibitors; however, as this is a single case report, these observations are hypothesis-generating and require validation in larger cohorts to determine any broader clinical relevance in advanced HGSOC. While this case is still based on only 2 CTC candidates and 1 putative CTC captured using molecular imaging methods, it provides an illustrative example of how single-cell and bulk RNA profiling of liquid biopsy components can inform our understanding of tumor–host interactions, support prognostic assessment, and potentially guide treatment decisions in real time, these insights warrant validation in larger patient cohorts to fully establish their clinical relevance.

## Data Availability

The datasets presented in this study can be found in online repositories. The names of the repository/repositories and accession number(s) can be found in the article/**Supplementary Material**.

## References

[1] Torre LA Trabert B DeSantis CE Miller KD Samimi G Runowicz CD . Ovarian cancer statistics, 2018. CA Cancer J Clin. (2018) 68:284–96. doi: 10.3322/caac.21456, PMID: 29809280 PMC6621554

[2] Kurman RJ Shih IM . Molecular pathogenesis and extraovarian origin of epithelial ovarian cancer. Shifting the paradigm. Hum Pathol. (2011) 42:918–31. doi: 10.1016/j.humpath.2011.03.003, PMID: 21683865 PMC3148026

[3] Bowtell DD Böhm S Ahmed AA Aspuria PJ Bast RC Beral V . Rethinking ovarian cancer II: reducing mortality from high-grade serous ovarian cancer. Nat Rev Cancer. (2015) 15:668–79. doi: 10.1038/nrc4019, PMID: 26493647 PMC4892184

[4] Nelson BH Hamilton P Phung MT Milne K Harris B Thornton S . Immunological and molecular features of the tumor microenvironment of long-term survivors of ovarian cancer. J Clin Invest. (2024) 134. Available online at: https://www.jci.org/articles/view/179501 (Accessed November 18, 2025)., PMID: 39470729 10.1172/JCI179501 PMC11645148

[5] Hoppenot C Eckert MA Tienda SM Lengyel E . Who are the long-term survivors of high grade serous ovarian cancer? Gynecol Oncol. (2018) 148:204–12. doi: 10.1016/j.ygyno.2017.10.032, PMID: 29128106

[6] Jochum F De Rozario T Lecointre L Faller E Boisrame T Dabi Y . Adherence to European ovarian cancer guidelines and impact on survival: a French multicenter study (FRANCOGYN). Int J Gynecol Cancer Off J Int Gynecol Cancer Soc. (2021) 31:1443–52. doi: 10.1136/ijgc-2021-002934, PMID: 34607855

[7] Goenka L Nakka T Dubashi B Kayal S Penumadu P Chaturvedula L . A simple, novel prognostic score in platinum sensitive relapsed ovarian cancer. Am J Clin Oncol. (2021) 44:434–41. doi: 10.1097/COC.0000000000000830, PMID: 34081031

[8] Wang H Liu J Yang J Wang Z Zhang Z Peng J . A novel tumor mutational burden-based risk model predicts prognosis and correlates with immune infiltration in ovarian cancer. Front Immunol. (2022) 13:943389. doi: 10.3389/fimmu.2022.943389, PMID: 36003381 PMC9393426

[9] Chase DM Mahajan A Scott DA Hawkins N Kalilani L . Correlation between progression-free survival and overall survival in patients with ovarian cancer after cytoreductive surgery: a systematic literature review. Int J Gynecol Cancer Off J Int Gynecol Cancer Soc. (2023) 33:1602–11. doi: 10.1136/ijgc-2023-004487, PMID: 37643825 PMC10579502

[10] Li Z Yin H Ren M Shen Y . Prognostic significance of CA125 dynamic change for progression free survival in patients with epithelial ovarian carcinoma. Med Sci Monit Int Med J Exp Clin Res. (2020) 26:e925051. doi: 10.12659/MSM.925051, PMID: 32908118 PMC7504865

[11] Stewart MD Merino Vega D Arend RC Baden JF Barbash O Beaubier N . Homologous recombination deficiency: concepts, definitions, and assays. Oncologist. (2022) 27:167–74. doi: 10.1093/oncolo/oyab053, PMID: 35274707 PMC8914493

[12] Royfman R Whiteley E Noe O Morand S Creeden J Stanbery L . BRCA1/2 signaling and homologous recombination deficiency in breast and ovarian cancer. Future Oncol. (2021) 17:2817–30. doi: 10.2217/fon-2021-0072, PMID: 34058833

[13] Jansaka N Suprasert P . Survival outcomes of recurrent epithelial ovarian cancer: experience from a Thailand northern tertiary care center. Asian Pac J Cancer Prev APJCP. (2014) 15:10837–40. doi: 10.7314/APJCP.2014.15.24.10837, PMID: 25605186

[14] Moore KN Pothuri B Monk B Coleman RL . PARP inhibition as frontline therapy in ovarian cancer. Clin Adv Hematol Oncol HO. (2020) 18:550–6. doi: 10.1016/j.ygyno.2017.10.032, PMID: 33006584

[15] Di Donato V Caruso G Golia D’Augè T Perniola G Palaia I Tomao F . Prognostic impact of microscopic residual disease after neoadjuvant chemotherapy in patients undergoing interval debulking surgery for advanced ovarian cancer. Arch Gynecol Obstet. (2025) 311:429–36. doi: 10.1007/s00404-024-07775-w, PMID: 39397086 PMC11890345

[16] Gammall J Lai AG . Prognostic determinants in cancer survival: a multidimensional evaluation of clinical and genetic factors across 10 cancer types in the participants of Genomics England’s 100,000 Genomes Project. Discov Oncol. (2024) 15:448. doi: 10.1007/s12672-024-01310-8, PMID: 39277826 PMC11402888

[17] Asante DB Calapre L Ziman M Meniawy TM Gray ES . Liquid biopsy in ovarian cancer using circulating tumor DNA and cells: Ready for prime time? Cancer Lett. (2020) 468:59–71. doi: 10.1016/j.canlet.2019.10.014, PMID: 31610267

[18] Zhu JW Charkhchi P Akbari MR . Potential clinical utility of liquid biopsies in ovarian cancer. Mol Cancer. (2022) 21:114. doi: 10.1186/s12943-022-01588-8, PMID: 35545786 PMC9092780

[19] Wenta R Richert J Muchlińska A Senkus E Suchodolska G Łapińska-Szumczyk S . Measurable morphological features of single circulating tumor cells in selected solid tumors—A pilot study. Cytometry A. (2024) 105:883–92. doi: 10.1002/cyto.a.24906, PMID: 39498617

[20] Muchlińska A Wenta R Ścińska W Markiewicz A Suchodolska G Senkus E . Improved characterization of circulating tumor cells and cancer-associated fibroblasts in one-tube assay in breast cancer patients using imaging flow cytometry. Cancers. (2023) 15:4169. doi: 10.3390/cancers15164169, PMID: 37627197 PMC10453498

[21] Best MG In ‘t Veld SGJG Sol N Wurdinger T . RNA sequencing and swarm intelligence–enhanced classification algorithm development for blood-based disease diagnostics using spliced blood platelet RNA. Nat Protoc. (2019) 14:1206–34. doi: 10.1038/s41596-019-0139-5, PMID: 30894694

[22] Franzén O Gan LM Björkegren JLM . PanglaoDB: a web server for exploration of mouse and human single-cell RNA sequencing data. Database. (2019) 2019:baz046. doi: 10.1093/database/baz046, PMID: 30951143 PMC6450036

[23] Zannoni GF Angelico G Spadola S Bragantini E Troncone G Fraggetta F . Chemotherapy Response Score (CRS): A comprehensive review of its prognostic and predictive value in High-Grade Serous Carcinoma (HGSC). Gynecol Oncol. (2025) 194:1–10. doi: 10.1016/j.ygyno.2025.01.012, PMID: 39919553

[24] Xie Q Liu S Zhang S Liao L Xiao Z Wang S . Research progress on the multi-omics and survival status of circulating tumor cells. Clin Exp Med. (2024) 24:49. doi: 10.1007/s10238-024-01309-z, PMID: 38427120 PMC10907490

[25] Zhou S Xu H Duan Y Tang Q Huang H Bi F . Survival mechanisms of circulating tumor cells and their implications for cancer treatment. Cancer Metastasis Rev. (2024) 43:941–57. doi: 10.1007/s10555-024-10178-7, PMID: 38436892

[26] Han Y . Chemoresistance and metastatic potential of ovarian cancer cells governed by the tumor microenvironment. Poland: Medical University of Gdańsk (2021). Available online at: https://s-space.snu.ac.kr/handle/10371/176476 (Accessed November 18, 2025).

[27] Segal BH Giridharan T Suzuki S Khan ANH Zsiros E Emmons TR . Neutrophil interactions with T cells, platelets, endothelial cells, and of course tumor cells. Immunol Rev. (2023) 314:13–35. doi: 10.1111/imr.13178, PMID: 36527200 PMC10174640

[28] Lou E Vogel RI Teoh D Hoostal S Grad A Gerber M . Assessment of circulating tumor cells as a predictive biomarker of histology in women with suspected ovarian cancer. Lab Med. (2018) 49:134–9. doi: 10.1093/labmed/lmx084, PMID: 29361118 PMC6251585

[29] Obermayr E Bednarz-Knoll N Orsetti B Weier HU Lambrechts S Castillo-Tong DC . Circulating tumor cells: potential markers of minimal residual disease in ovarian cancer? a study of the OVCAD consortium. Oncotarget. (2017) 8:106415–28. doi: 10.18632/oncotarget.22468, PMID: 29290959 PMC5739744

[30] Jopek MA Sieczczyński M Pastuszak K Łapińska-Szumczyk S Jassem J Żaczek AJ . Impact of clinical factors on accuracy of ovarian cancer detection via platelet RNA profiling. Blood Adv. (2024) 9:979–89. doi: 10.1182/bloodadvances.2024014008, PMID: 39715465 PMC11907454

[31] Wu B Zhang B Li B Wu H Jiang M . Cold and hot tumors: from molecular mechanisms to targeted therapy. Signal Transduct Target Ther. (2024) 9:274. doi: 10.1038/s41392-024-01979-x, PMID: 39420203 PMC11491057

[32] Zhang D Wang W Tang M Qu C Jiang Z Li X . *In situ* gene engineering approach to overcome tumor resistance and enhance T cell-mediated cancer immunotherapy. Nano Lett. (2025) 25:6200–8. doi: 10.1021/acs.nanolett.5c00488, PMID: 40180619

[33] Pastuszak K Supernat A Best MG In ‘t Veld SGJG Łapińska-Szumczyk S Łojkowska A . imPlatelet classifier: image-converted RNA biomarker profiles enable blood-based cancer diagnostics. Mol Oncol. (2021) 15:2688–701. doi: 10.1002/1878-0261.13014, PMID: 34013585 PMC8486571

[34] Zuckerbrot-Schuldenfrei M Aviel-Ronen S Zilberberg A Efroni S . Ovarian cancer is detectable from peripheral blood using machine learning over T-cell receptor repertoires. Brief Bioinform. (2024) 25:bbae075. doi: 10.1093/bib/bbae075, PMID: 38483254 PMC10938541

