# Supplementary material for: Case Report: Blood single-cell analysis of a IVB high-grade serous ovarian cancer patient presenting a favorable prognosis
Source: Front Oncol. 2025 Nov 24;15:1697863. doi: 10.3389/fonc.2025.1697863 (PMC12682629; doi:10.3389/fonc.2025.1697863)
Supplement: Supplementary file 1 [file DataSheet1.docx]

**SUPPLEMENTARY MATERIAL**


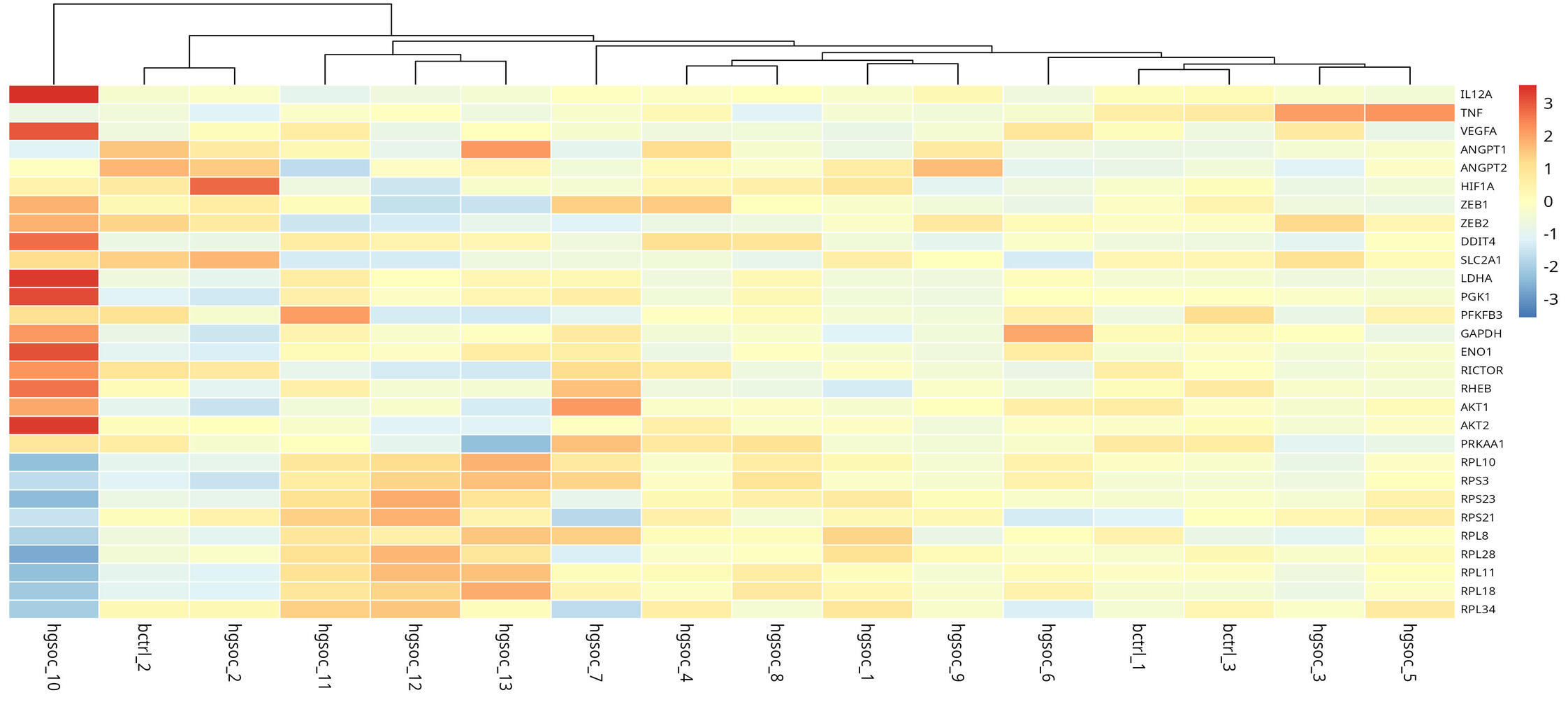
**Figure S1** A heatmap of selected genes for **B-cells**. The values are z-scores of CPM-normalized pseudobulk. The case patient’s ID is hgsoc_10.


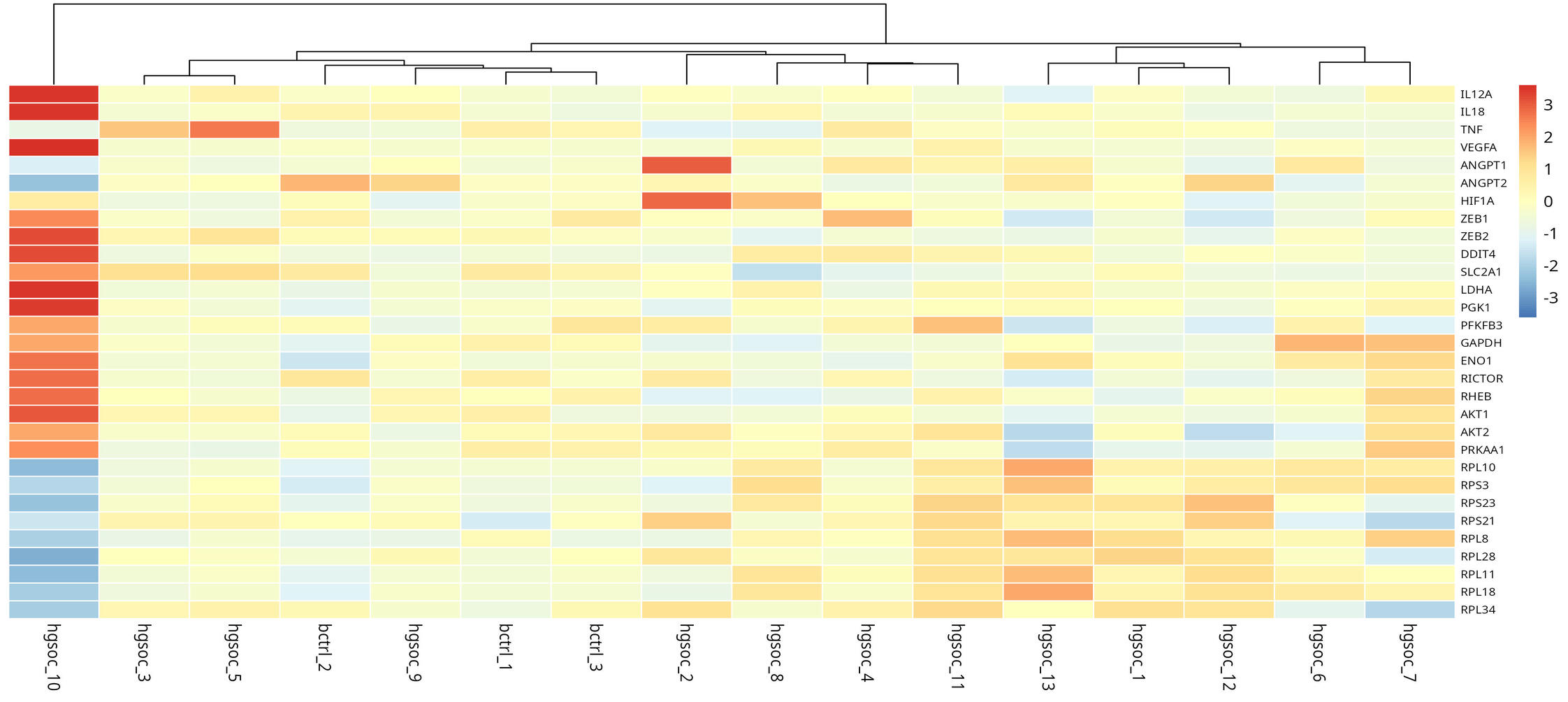
**Figure S2** A heatmap of selected genes for **T cells.** The values are z-scores of CPM-normalized pseudobulk. The case patient’s ID is hgsoc_10.


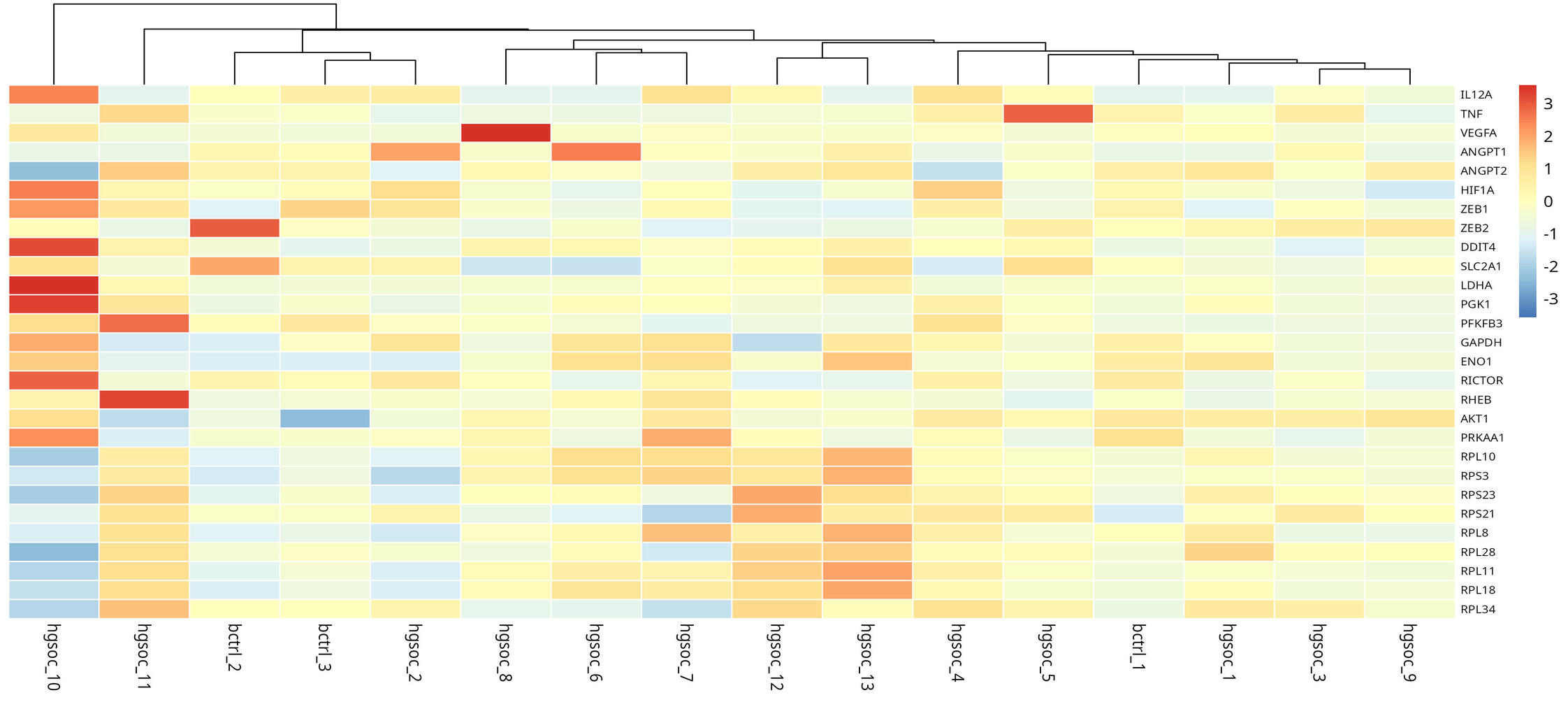
**Figure S3** A heatmap of selected genes for **NK-cells**. The values are z-scores of CPM-normalized pseudobulk. The case patient’s ID is hgsoc_10.


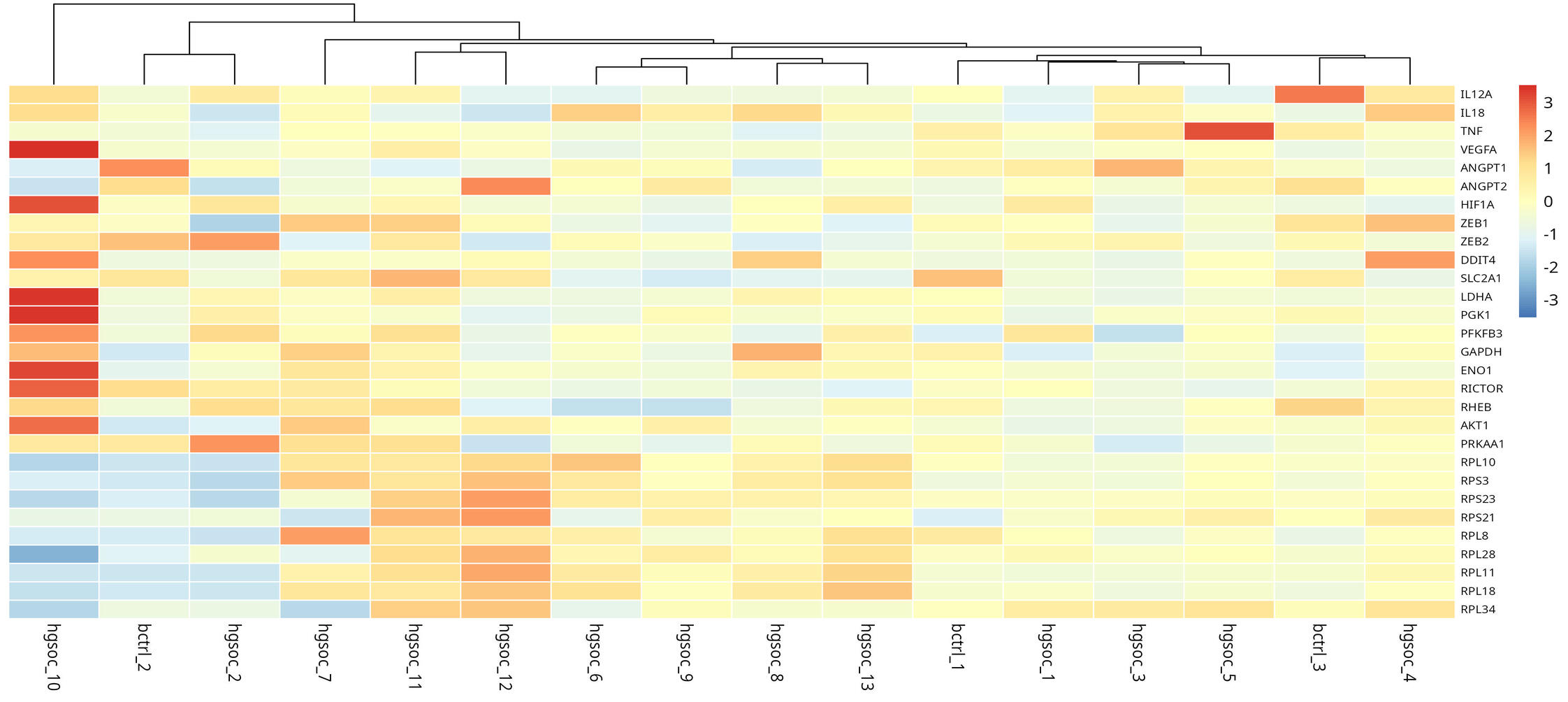
**Figure S4** Heatmap of selected genes for **monocytes**. The values are z-scores of CPM-normalized pseudobulk. The case patient’s ID is hgsoc_10.

**Table S1** List of markers used for cell type classification in single cell RNA sequencing.

| **Cell type** | **List of Markers** |
| --- | --- |
| B Cells | MS4A1, CD19 |
| CD8+ T Cells | CD8A, CD8B |
| NK Cells | KLRF1,NCAM1 |
| Macrophages | CD68 |
| Monocytes | FCN1, LYZ, FCGR3B, S100A9, CD14, FCGR3A |
| CD4+ T Cells | CD4, IL7R (17,29) |
| Memory T Cells | SELL, CCR7 |
| Memory B Cells | CD27 |
| Regulatory T Cells | IL2RA, FOXP3, TIGIT(17,29) |
| Fibroblasts | PDGFRB, FAP |
| Platelets | PPBP, ITGB3, PF4, GP9, ITGA2B, CD63, SELP |
| Dendritic Cells | FCER1A, CD1C, AXL, CD83 |
| Neutrophils | FUT4, CEACAM8, ITGAM, ITGB2 |
| Epithelial CTCs | KRT7, CLDN4, EPCAM, SDC4, SDC1, PERP, APP, EMP2, LMNA, DSP, TSPAN13, SLC39A7, ADAM15, EPPK1, CD59, ABHD11, PRSS8, NQO1, KDELR2, HID1, EFNA1, KDELR1, TSC22D1, ENAH, KIAA1522, DHCR24, COPG1, RAE1, TACSTD2, ERBB3, NANS, MLPH, LAMA5, TRAF4, RAB25, NPDC1, KIAA1217, SLC39A1, PDXDC1, CYTH2, MGST3, EFHD1, EIF4G1, TMED9, TMEM9, CSRP1, CHCHD2, RHOB, P4HB, SPDEF, MISP, SURF4, NME1, CDK4, APH1A, ENTPD6, PTPRS, SCARB2, STAU1, ESRP1, DAG1, TUBA1C, LRRC59, MAL2, TCEAL4, BAIAP2, SLC50A1, TFAP2C, EHF, CDK16, NELFCD, AAMP, PAFAH1B3, ILF2, CLTA, WDR34, PTPRF, RHOV, BANF1, MYO5C, AP1M2, TJP3, CCT6A, YIF1A, GSN, DUSP14, PGRMC1, EPS8L1, TMEM147, RPN2, PHB, TRPS1, S100A13, SRGAP1, MRPS26, ARPC1A, CCT5 |
| Mesenchymal CTCs | HMGA1, LMNA, CD9, TUBA1C, CD59, PYGB, VAT1, RAB34,  DSTN, DNAJC9, VIM, EDARADD, RHOC, TUBB, PKM, ENO1, TUBB4B, EIF6, ELK3, GLUD1, GLUD2, S100A16, ATL3, CYB561, S100A6, TRAM2, RPL7L1, S100A10, PRSS23, CNN3, PFKP, CAPN2, ADRM1, TAX1BP3, CALU, NME2, UBE2E3, GAPDH, CTTN, GSS, EIF4G1, SLC39A7, SLC39A13, ITGA6, TPM4, ACTN4, AXL, TXN, BCL2L1, YWHAQ, LDLR, OCIAD2, FLNB, TPD52L2, NT5E, AK1, POLR2L, CD63, TIMM23, PLP2, PIGT, PSMC3, ITGA3, ANAPC11, MAP1B, TNFRSF12A, TPI1, SPATS2L, ATP1A1, RAC1, PMP22, VKORC1, DST, SMURF2, LRRC8A, DBNDD2, MRPL4, ARPC1A, CLIC4, ANXA5, FN1, CAV1 |

List of markers used for identifying epithelial, mesenchymal and platelets-cloaked CTCs.(21)

**Table S2** Comparison between mean of pseudobulk, CPM-normalized expression of others ovarian cancer patients and case report patient. The pseudobulk was created by aggregating **B-cells** from the single cell dataset. The rows were colored according to gene functions in the following way: blue - ribosome-related, orange - VEGF-related, green - angiogenesis-related, gray - mTOR-related, purple - hypoxia-related, pink - glycolysis-related, red - inflammatory-related, brown - lipid-related

|  | Mean of other OC patients | Case report patient |
| --- | --- | --- |
| RPL18A | 3015.71 | 843.41 |
| RPS4X | 4331.55 | 2412.76 |
| RPL10 | 6398.74 | 1936.28 |
| RPS3 | 3749.24 | 1607.63 |
| RPS23 | 4174.71 | 1096.83 |
| RPS21 | 1568.60 | 754.98 |
| RPL8 | 2173.62 | 943.72 |
| RPL28 | 3062.91 | 946.36 |
| RPS8 | 4026.97 | 1326.49 |
| RPS7 | 2300.67 | 935.80 |
| RPS14 | 4790.20 | 1399.09 |
| RPL30 | 4321.26 | 1222.22 |
| RPL13 | 6673.14 | 2010.19 |
| RPL5 | 2491.70 | 1759.42 |
| RPL11 | 4047.64 | 1271.06 |
| RPL10A | 1994.25 | 727.26 |
| RPLP2 | 2300.40 | 929.20 |
| RPL18 | 3060.26 | 886.97 |
| RPL19 | 3236.25 | 1059.87 |
| RPL9 | 2790.24 | 1150.95 |
| RPS6 | 3735.43 | 1630.07 |
| RPL37A | 1415.84 | 941.08 |
| RPS5 | 1757.14 | 558.31 |
| RPL27 | 1165.40 | 487.04 |
| RPS25 | 1931.97 | 685.02 |
| RPL22L1 | 109.52 | 60.72 |
| RPS19 | 2921.47 | 906.77 |
| RPLP0 | 1395.48 | 1022.92 |
| RPS18 | 5297.15 | 1680.22 |
| RPL27A | 1797.28 | 819.65 |
| RPL22 | 1504.17 | 662.59 |
| RPL24 | 1690.87 | 685.02 |
| RPL15 | 2409.96 | 1437.36 |
| RPS11 | 1577.87 | 748.38 |
| RPS13 | 2347.96 | 642.79 |
| RPL12 | 2556.64 | 840.77 |
| RPS28 | 3531.01 | 1453.20 |
| RPS24 | 2353.94 | 888.29 |
| RPL14 | 2045.14 | 703.50 |
| RPL34 | 3525.67 | 1290.85 |
| RPL7A | 2916.83 | 1789.77 |
| RPS15 | 2050.69 | 547.75 |
| RPL23 | 971.84 | 409.17 |
| RPS9 | 2230.31 | 630.91 |
| RPS27 | 4324.47 | 2581.71 |
| RPS12 | 5678.11 | 1413.60 |
| RPL35 | 1214.48 | 524.00 |
| RPS20 | 1188.10 | 650.71 |
| RPL32 | 3990.12 | 1094.19 |
| RPL4 | 688.58 | 698.22 |
| RPL36AL | 559.56 | 161.03 |
| RPSA | 1759.18 | 1212.98 |
| RPL39 | 3581.12 | 1463.76 |
| RPS16 | 2252.66 | 756.30 |
| RPL37 | 2199.03 | 1179.98 |
| RPL3 | 3716.79 | 5410.24 |
| RPL36 | 1399.40 | 559.63 |
| RPS2 | 4887.19 | 3179.62 |
| RPL7 | 1238.66 | 655.99 |
| RPL6 | 1876.32 | 997.84 |
| RPL23A | 2114.45 | 952.96 |
| RPL29 | 2334.50 | 681.06 |
| RPS27A | 4073.88 | 1145.67 |
| RPL13A | 2735.49 | 1804.29 |
| RPS27L | 128.90 | 69.95 |
| RPS26 | 443.57 | 109.55 |
| RPL35A | 2058.49 | 674.46 |
| RPS3A | 4078.92 | 1818.81 |
| VEGFA | 0.46 | 2.64 |
| VEGFB | 42.66 | 60.72 |
| VEGFC | 0.01 | 0.00 |
| VEGFD | 0.06 | 0.00 |
| FLT1 | 4.94 | 1.32 |
| FLT4 | 0.68 | 0.00 |
| ANGPT1 | 1.21 | 0.00 |
| ANGPT2 | 38.29 | 38.28 |
| PDGFB | 0.89 | 0.00 |
| TEK | 0.17 | 0.00 |
| FGF2 | 0.51 | 1.32 |
| PECAM1 | 44.91 | 43.56 |
| MTOR | 43.33 | 40.92 |
| RPTOR | 103.09 | 112.19 |
| RICTOR | 267.90 | 442.16 |
| RHEB | 61.35 | 95.03 |
| AKT1 | 19.98 | 27.72 |
| AKT2 | 30.89 | 75.23 |
| AKT3 | 324.26 | 380.13 |
| PRKAA1 | 57.74 | 68.63 |
| PRKAA2 | 0.02 | 0.00 |
| HIF1A | 182.14 | 221.74 |
| ZEB1 | 302.09 | 468.56 |
| ZEB2 | 286.83 | 642.79 |
| DDIT4 | 51.49 | 146.51 |
| PIM1 | 60.89 | 40.92 |
| BNIP3 | 16.20 | 9.24 |
| SLC2A1 | 23.74 | 38.28 |
| LDHA | 118.92 | 380.13 |
| 1 PGK | 129.82 | 252.10 |
| HK2 | 6.04 | 5.28 |
| PFKFB3 | 29.94 | 39.60 |
| SLC16A1 | 24.77 | 48.84 |
| GAPDH | 314.94 | 617.71 |
| ENO1 | 146.54 | 318.09 |
| PKM | 70.02 | 117.47 |
| MYC | 47.71 | 18.48 |
| PDK1 | 66.42 | 79.19 |
| SLC16A2 | 0.14 | 0.00 |
| SLC16A3 | 16.95 | 42.24 |
| CSF1R | 1.33 | 5.28 |
| IL12A | 1.32 | 9.24 |
| TNF | 11.95 | 5.28 |
| CSF3R | 3.29 | 6.60 |
| IL18 | 3.42 | 5.28 |
| IL6 | 2.29 | 3.96 |
| CSF2RB | 4.24 | 9.24 |
| CSF2RA | 1.71 | 1.32 |
| CSF1 | 0.97 | 1.32 |
| IL1B | 0.72 | 0.00 |
| IFNG | 9.42 | 23.76 |
| APOE | 0.15 | 0.00 |
| APOC1 | 0.06 | 0.00 |
| APOC2 | 0.04 | 0.00 |
| SORL1 | 175.35 | 149.15 |
| ABCA7 | 16.51 | 21.12 |
| PSEN1 | 92.83 | 117.47 |
| PSEN2 | 2.15 | 3.96 |
| APP | 73.55 | 54.12 |
| TMEM106B | 37.26 | 27.72 |
| GRN | 20.15 | 51.48 |
| ABCC9 | 0.08 | 1.32 |
| KCNMB2 | 0.93 | 1.32 |
| APOA1 | 0.30 | 0.00 |
| APOC1.1 | 0.06 | 0.00 |
| APOC2.1 | 0.04 | 0.00 |
| LDLR | 14.46 | 18.48 |
| ABCA1 | 60.63 | 35.64 |
| CETP | 0.84 | 0.00 |
| HMGCR | 12.79 | 19.80 |
| SRARB1 | 0.00 | 0.00 |
| PLTP | 0.56 | 1.32 |
| APOA5 | 0.03 | 0.00 |
| CYP27A1 | 0.92 | 0.00 |
| SORT1 | 1.16 | 6.60 |

**Table S3** Comparison between mean of pseudobulk, CPM-normalized expression of others ovarian cancer patients and case report patient. The pseudobulk was created by aggregating **T-cells** from the single cell dataset. The rows were colored according to gene functions in the following way: blue - ribosome-related, orange - VEGF-related, green - angiogenesis-related, gray - mTOR-related, purple - hypoxia-related, pink - glycolysis-related, red - inflammatory-related, brown - lipid-related

|  | Mean of other OC patients | Case report patient |
| --- | --- | --- |
| RPL18A | 2825.17 | 850.60 |
| RPS4X | 4814.97 | 2588.05 |
| RPL10 | 7096.79 | 2177.15 |
| RPS3 | 4392.29 | 2014.90 |
| RPS23 | 4184.05 | 1237.33 |
| RPS21 | 1704.05 | 832.85 |
| RPL8 | 2051.98 | 847.77 |
| RPL28 | 3353.47 | 1040.62 |
| RPS8 | 4104.72 | 1287.21 |
| RPS7 | 2380.54 | 1019.79 |
| RPS14 | 5614.12 | 1684.99 |
| RPL30 | 4767.63 | 1450.49 |
| RPL13 | 7305.89 | 2499.59 |
| RPL5 | 2714.88 | 2151.43 |
| RPL11 | 4295.27 | 1338.12 |
| RPL10A | 2077.63 | 756.23 |
| RPLP2 | 2466.99 | 974.02 |
| RPL18 | 3177.77 | 928.25 |
| RPL19 | 3400.04 | 1138.07 |
| RPL9 | 3074.08 | 1330.66 |
| RPS6 | 4143.06 | 1801.73 |
| RPL37A | 1464.16 | 926.97 |
| RPS5 | 1677.38 | 456.15 |
| RPL27 | 1247.69 | 530.21 |
| RPS25 | 2153.60 | 738.49 |
| RPL22L1 | 79.57 | 49.88 |
| RPS19 | 3083.08 | 1000.25 |
| RPLP0 | 1445.80 | 1073.53 |
| RPS18 | 5703.56 | 1596.28 |
| RPL27A | 1935.07 | 925.17 |
| RPL22 | 1622.28 | 751.60 |
| RPL24 | 1808.93 | 768.57 |
| RPL15 | 2349.70 | 1550.77 |
| RPS11 | 1382.66 | 522.24 |
| RPS13 | 2522.91 | 697.35 |
| RPL12 | 2561.16 | 745.69 |
| RPS28 | 3915.56 | 1546.14 |
| RPS24 | 2433.33 | 955.51 |
| RPL14 | 2351.34 | 884.02 |
| RPL34 | 4004.47 | 1550.00 |
| RPL7A | 2973.52 | 1730.76 |
| RPS15 | 2135.51 | 607.61 |
| RPL23 | 1029.62 | 415.53 |
| RPS9 | 2290.14 | 674.97 |
| RPS27 | 4618.07 | 3020.80 |
| RPS12 | 6808.77 | 1819.73 |
| RPL35 | 1230.16 | 514.27 |
| RPS20 | 1264.63 | 666.23 |
| RPL32 | 4342.20 | 1308.55 |
| RPL4 | 751.15 | 744.40 |
| RPL36AL | 644.63 | 219.33 |
| RPSA | 1853.25 | 1416.55 |
| RPL39 | 3864.98 | 1493.43 |
| RPS16 | 2446.29 | 799.17 |
| RPL37 | 2358.89 | 1282.58 |
| RPL3 | 4117.64 | 6334.48 |
| RPL36 | 1539.36 | 576.24 |
| RPS2 | 5053.20 | 3242.71 |
| RPL7 | 1361.57 | 832.60 |
| RPL6 | 2077.48 | 1186.41 |
| RPL23A | 2230.55 | 971.19 |
| RPL29 | 2349.30 | 641.55 |
| RPS27A | 4608.88 | 1326.81 |
| RPL13A | 2791.95 | 1929.01 |
| RPS27L | 131.22 | 60.68 |
| RPS26 | 496.02 | 117.00 |
| RPL35A | 2242.02 | 836.97 |
| RPS3A | 4515.27 | 2169.69 |
| VEGFA | 0.23 | 2.31 |
| VEGFB | 30.00 | 27.26 |
| VEGFC | 0.04 | 0.00 |
| VEGFD | 0.01 | 0.00 |
| FLT1 | 1.95 | 2.57 |
| FLT4 | 1.24 | 0.26 |
| ANGPT1 | 1.19 | 0.00 |
| ANGPT2 | 35.43 | 20.31 |
| PDGFB | 2.21 | 1.54 |
| TEK | 0.15 | 0.00 |
| FGF2 | 0.07 | 0.00 |
| PECAM1 | 34.01 | 47.83 |
| MTOR | 42.34 | 32.40 |
| RPTOR | 121.96 | 130.37 |
| RICTOR | 288.47 | 562.86 |
| RHEB | 65.59 | 104.14 |
| AKT1 | 21.34 | 38.06 |
| AKT2 | 28.73 | 38.31 |
| AKT3 | 462.10 | 546.67 |
| PRKAA1 | 67.21 | 101.31 |
| PRKAA2 | 0.04 | 0.00 |
| HIF1A | 140.88 | 180.51 |
| ZEB1 | 327.90 | 617.89 |
| ZEB2 | 196.56 | 753.91 |
| DDIT4 | 71.86 | 284.13 |
| PIM1 | 113.22 | 70.20 |
| BNIP3 | 22.55 | 13.89 |
| SLC2A1 | 14.26 | 24.68 |
| LDHA | 154.51 | 412.96 |
| 1 PGK | 148.43 | 273.59 |
| HK2 | 4.50 | 2.83 |
| PFKFB3 | 44.85 | 79.45 |
| SLC16A1 | 26.71 | 29.83 |
| GAPDH | 308.27 | 467.47 |
| ENO1 | 165.89 | 251.73 |
| PKM | 75.02 | 78.94 |
| MYC | 61.81 | 27.51 |
| PDK1 | 90.57 | 70.97 |
| SLC16A2 | 0.47 | 0.51 |
| SLC16A3 | 18.89 | 45.51 |
| CPT1A | 51.52 | 65.83 |
| GLS | 297.74 | 388.79 |
| ACLY | 20.62 | 33.94 |
| CSF1R | 1.80 | 3.09 |
| IL12A | 0.51 | 2.57 |
| TNF | 22.10 | 6.94 |
| CSF3R | 4.50 | 11.83 |
| IL18 | 3.11 | 8.49 |
| IL6 | 0.15 | 0.00 |
| CSF2RB | 1.35 | 5.40 |
| CSF2RA | 3.02 | 9.26 |
| CSF1 | 1.82 | 1.54 |
| IL1B | 1.11 | 3.34 |
| IFNG | 18.63 | 29.31 |
| APOE | 0.17 | 0.00 |
| APOC1 | 0.03 | 0.00 |
| APOC2 | 0.00 | 0.26 |
| SORL1 | 310.20 | 357.42 |
| ABCA7 | 13.91 | 11.83 |
| PSEN1 | 82.72 | 91.28 |
| PSEN2 | 1.05 | 2.31 |
| APP | 59.63 | 78.68 |
| TMEM106B | 47.37 | 49.63 |
| GRN | 12.32 | 29.57 |
| ABCC9 | 0.18 | 1.80 |
| KCNMB2 | 0.77 | 1.03 |
| APOA1 | 0.43 | 0.00 |
| APOC1.1 | 0.03 | 0.00 |
| APOC2.1 | 0.00 | 0.26 |
| LDLR | 18.88 | 23.66 |
| ABCA1 | 22.90 | 10.29 |
| CETP | 0.73 | 0.00 |
| HMGCR | 13.77 | 23.40 |
| SRARB1 | 0.00 | 0.00 |
| PLTP | 0.37 | 0.26 |
| APOA5 | 0.00 | 0.00 |
| CYP27A1 | 0.99 | 1.29 |
| SORT1 | 1.75 | 3.34 |

**Table S4** Comparison between mean of pseudobulk, CPM-normalized expression of others ovarian cancer patients and case report patient. The pseudobulk was created by aggregating **NK-cells** from the single cell dataset. The rows were colored according to gene functions in the following way: blue - ribosome-related, orange - VEGF-related, green - angiogenesis-related, gray - mTOR-related, purple - hypoxia-related, pink - glycolysis-related, red - inflammatory-related, brown - lipid-related

|  | Mean of other OC patients | Case report patient |
| --- | --- | --- |
| RPL18A | 1223.95 | 550.97 |
| RPS4X | 2136.44 | 1527.69 |
| RPL10 | 3547.07 | 1419.16 |
| RPS3 | 2434.76 | 1318.99 |
| RPS23 | 1936.46 | 701.23 |
| RPS21 | 799.00 | 550.97 |
| RPL8 | 992.56 | 617.75 |
| RPL28 | 1602.29 | 705.41 |
| RPS8 | 1560.53 | 780.54 |
| RPS7 | 1409.79 | 834.80 |
| RPS14 | 2715.68 | 1214.64 |
| RPL30 | 2130.23 | 868.19 |
| RPL13 | 3085.94 | 1277.25 |
| RPL5 | 1280.47 | 1306.46 |
| RPL11 | 1820.01 | 830.63 |
| RPL10A | 942.35 | 413.23 |
| RPLP2 | 1208.02 | 546.79 |
| RPL18 | 1507.68 | 634.45 |
| RPL19 | 1686.94 | 759.67 |
| RPL9 | 1284.08 | 672.01 |
| RPS6 | 1755.51 | 914.11 |
| RPL37A | 797.30 | 692.88 |
| RPS5 | 724.15 | 292.18 |
| RPL27 | 708.14 | 358.96 |
| RPS25 | 921.11 | 450.79 |
| RPL22L1 | 66.47 | 16.70 |
| RPS19 | 1742.12 | 788.89 |
| RPLP0 | 593.20 | 621.93 |
| RPS18 | 2373.73 | 939.15 |
| RPL27A | 1030.66 | 763.84 |
| RPL22 | 706.64 | 450.79 |
| RPL24 | 953.92 | 563.49 |
| RPL15 | 1412.90 | 964.20 |
| RPS11 | 689.73 | 379.83 |
| RPS13 | 1007.67 | 346.44 |
| RPL12 | 1165.41 | 450.79 |
| RPS28 | 1806.84 | 993.41 |
| RPS24 | 1674.78 | 834.80 |
| RPL14 | 1239.58 | 634.45 |
| RPL34 | 1728.96 | 759.67 |
| RPL7A | 1692.14 | 1181.24 |
| RPS15 | 1118.04 | 421.57 |
| RPL23 | 359.32 | 192.00 |
| RPS9 | 1143.10 | 425.75 |
| RPS27 | 2323.07 | 2011.87 |
| RPS12 | 2627.83 | 1156.20 |
| RPL35 | 725.93 | 375.66 |
| RPS20 | 529.65 | 313.05 |
| RPL32 | 1760.64 | 788.89 |
| RPL4 | 360.67 | 484.18 |
| RPL36AL | 479.29 | 204.53 |
| RPSA | 865.45 | 960.02 |
| RPL39 | 1548.37 | 934.98 |
| RPS16 | 1045.05 | 500.88 |
| RPL37 | 1137.90 | 830.63 |
| RPL3 | 2301.28 | 4098.87 |
| RPL36 | 648.79 | 375.66 |
| RPS2 | 2376.64 | 1970.13 |
| RPL7 | 601.98 | 471.66 |
| RPL6 | 1142.60 | 830.63 |
| RPL23A | 1265.63 | 646.97 |
| RPL29 | 1188.06 | 550.97 |
| RPS27A | 2440.22 | 797.24 |
| RPL13A | 1384.25 | 1231.33 |
| RPS27L | 125.06 | 58.44 |
| RPS26 | 359.93 | 141.92 |
| RPL35A | 1019.61 | 563.49 |
| RPS3A | 2003.54 | 1239.68 |
| VEGFA | 1.66 | 4.17 |
| VEGFB | 26.58 | 33.39 |
| VEGFC | 0.03 | 0.00 |
| VEGFD | 0.07 | 0.00 |
| FLT1 | 01.09 | 4.17 |
| FLT4 | 0.19 | 0.00 |
| ANGPT1 | 0.63 | 0.00 |
| ANGPT2 | 38.79 | 16.70 |
| PDGFB | 01.06 | 0.00 |
| TEK | 0.13 | 0.00 |
| FGF2 | 0.21 | 0.00 |
| PECAM1 | 42.30 | 29.22 |
| MTOR | 55.24 | 54.26 |
| RPTOR | 129.96 | 87.65 |
| RICTOR | 372.15 | 646.97 |
| RHEB | 97.41 | 104.35 |
| AKT1 | 41.93 | 50.09 |
| AKT2 | 45.63 | 37.57 |
| AKT3 | 610.42 | 538.45 |
| PRKAA1 | 91.78 | 133.57 |
| PRKAA2 | 0.03 | 0.00 |
| HIF1A | 135.82 | 262.96 |
| ZEB1 | 161.86 | 300.53 |
| ZEB2 | 1107.71 | 1248.03 |
| DDIT4 | 139.71 | 442.44 |
| PIM1 | 62.39 | 16.70 |
| BNIP3 | 15.36 | 4.17 |
| SLC2A1 | 24.11 | 33.39 |
| LDHA | 136.73 | 429.92 |
| 1 PGK | 128.39 | 275.48 |
| HK2 | 1.37 | 0.00 |
| PFKFB3 | 38.69 | 62.61 |
| SLC16A1 | 21.06 | 4.17 |
| GAPDH | 309.28 | 396.53 |
| ENO1 | 156.75 | 196.18 |
| PKM | 48.55 | 29.22 |
| MYC | 6.27 | 4.17 |
| PDK1 | 38.88 | 29.22 |
| SLC16A2 | 0.30 | 0.00 |
| SLC16A3 | 47.04 | 96.00 |
| CPT1A | 111.63 | 91.83 |
| GLS | 338.14 | 425.75 |
| ACLY | 28.86 | 33.39 |
| CSF1R | 1.24 | 0.00 |
| IL12A | 0.92 | 4.17 |
| TNF | 25.07 | 8.35 |
| CSF3R | 3.65 | 16.70 |
| IL18 | 16.58 | 4.17 |
| IL6 | 0.05 | 0.00 |
| CSF2RB | 0.21 | 0.00 |
| CSF2RA | 1.68 | 4.17 |
| CSF1 | 0.93 | 4.17 |
| IL1B | 0.24 | 0.00 |
| IFNG | 56.12 | 29.22 |
| APOE | 0.13 | 0.00 |
| APOC1 | 0.04 | 0.00 |
| APOC2 | 0.46 | 0.00 |
| SORL1 | 403.89 | 555.14 |
| ABCA7 | 16.89 | 0.00 |
| PSEN1 | 93.64 | 121.05 |
| PSEN2 | 5.84 | 4.17 |
| APP | 13.30 | 8.35 |
| TMEM106B | 40.40 | 12.52 |
| GRN | 12.32 | 12.52 |
| ABCC9 | 0.01 | 0.00 |
| KCNMB2 | 0.66 | 0.00 |
| APOA1 | 0.30 | 0.00 |
| APOC1.1 | 0.04 | 0.00 |
| APOC2.1 | 0.46 | 0.00 |
| LDLR | 17.94 | 29.22 |
| ABCA1 | 10.20 | 0.00 |
| CETP | 0.85 | 0.00 |
| HMGCR | 23.57 | 41.74 |
| SRARB1 | 0.00 | 0.00 |
| PLTP | 1.16 | 0.00 |
| APOA5 | 0.00 | 0.00 |
| CYP27A1 | 1.64 | 4.17 |
| SORT1 | 2.28 | 0.00 |

**Table S5** Comparison between mean of pseudobulk, CPM-normalized expression of others ovarian cancer patients and case report patient. The pseudobulk was created by aggregating **Monocytes** from the single cell dataset. The rows were colored according to gene functions in the following way: blue - ribosome-related, orange - VEGF-related, green - angiogenesis-related, gray - mTOR-related, purple - hypoxia-related, pink - glycolysis-related, red - inflammatory-related, brown - lipid-related

|  | Mean of other OC patients | Case report patient |
| --- | --- | --- |
| RPL18A | 1299.73 | 564.29 |
| RPS4X | 1660.81 | 1161.19 |
| RPL10 | 2675.04 | 1259.86 |
| RPS3 | 1361.41 | 764.28 |
| RPS23 | 1773.85 | 723.46 |
| RPS21 | 622.80 | 439.78 |
| RPL8 | 1128.26 | 710.54 |
| RPL28 | 1765.37 | 829.72 |
| RPS8 | 1734.35 | 855.56 |
| RPS7 | 1134.44 | 623.16 |
| RPS14 | 2517.39 | 1070.74 |
| RPL30 | 1777.71 | 824.18 |
| RPL13 | 2768.21 | 1322.42 |
| RPL5 | 965.97 | 971.87 |
| RPL11 | 1741.74 | 940.28 |
| RPL10A | 735.97 | 376.40 |
| RPLP2 | 1174.76 | 626.85 |
| RPL18 | 1405.58 | 605.11 |
| RPL19 | 1450.94 | 717.72 |
| RPL9 | 1268.18 | 819.26 |
| RPS6 | 1501.19 | 845.31 |
| RPL37A | 832.52 | 692.29 |
| RPS5 | 509.77 | 210.87 |
| RPL27 | 685.96 | 446.14 |
| RPS25 | 724.75 | 401.01 |
| RPL22L1 | 64.58 | 36.10 |
| RPS19 | 1418.95 | 599.98 |
| RPLP0 | 649.32 | 552.19 |
| RPS18 | 2004.25 | 815.56 |
| RPL27A | 936.06 | 552.19 |
| RPL22 | 813.43 | 550.34 |
| RPL24 | 895.26 | 525.32 |
| RPL15 | 1361.30 | 1169.40 |
| RPS11 | 1034.08 | 605.11 |
| RPS13 | 1281.58 | 562.44 |
| RPL12 | 1196.32 | 540.09 |
| RPS28 | 1799.68 | 1067.04 |
| RPS24 | 1942.39 | 980.28 |
| RPL14 | 865.80 | 495.57 |
| RPL34 | 1712.15 | 999.56 |
| RPL7A | 1354.51 | 1056.79 |
| RPS15 | 1033.05 | 405.73 |
| RPL23 | 630.06 | 407.58 |
| RPS9 | 1661.78 | 819.87 |
| RPS27 | 1410.78 | 1240.99 |
| RPS12 | 2189.36 | 813.10 |
| RPL35 | 602.16 | 351.37 |
| RPS20 | 575.74 | 366.96 |
| RPL32 | 1691.55 | 763.87 |
| RPL4 | 427.49 | 461.93 |
| RPL36AL | 340.17 | 171.48 |
| RPSA | 511.25 | 477.11 |
| RPL39 | 1994.15 | 1100.48 |
| RPS16 | 1099.72 | 573.52 |
| RPL37 | 1105.01 | 834.03 |
| RPL3 | 1221.52 | 2143.11 |
| RPL36 | 647.00 | 364.09 |
| RPS2 | 2343.54 | 2058.81 |
| RPL7 | 848.07 | 660.29 |
| RPL6 | 1116.08 | 831.77 |
| RPL23A | 801.27 | 484.70 |
| RPL29 | 1163.76 | 523.27 |
| RPS27A | 1575.08 | 649.01 |
| RPL13A | 1224.34 | 949.10 |
| RPS27L | 122.01 | 56.41 |
| RPS26 | 271.57 | 93.54 |
| RPL35A | 998.55 | 599.16 |
| RPS3A | 1731.18 | 1174.73 |
| VEGFA | 7.98 | 35.08 |
| VEGFB | 8.34 | 8.00 |
| VEGFC | 0.16 | 0.62 |
| VEGFD | 0.08 | 0.41 |
| FLT1 | 2.60 | 02.05 |
| FLT4 | 0.25 | 0.00 |
| ANGPT1 | 14.23 | 5.13 |
| ANGPT2 | 8.14 | 3.69 |
| PDGFB | 0.35 | 0.00 |
| TEK | 0.20 | 0.00 |
| FGF2 | 0.20 | 0.00 |
| PECAM1 | 363.89 | 338.25 |
| MTOR | 41.18 | 30.15 |
| RPTOR | 93.37 | 91.28 |
| RICTOR | 227.32 | 379.48 |
| RHEB | 108.73 | 136.20 |
| AKT1 | 32.41 | 52.51 |
| AKT2 | 28.16 | 29.13 |
| AKT3 | 156.93 | 129.84 |
| PRKAA1 | 52.00 | 61.74 |
| PRKAA2 | 0.02 | 0.21 |
| HIF1A | 429.02 | 1201.19 |
| ZEB1 | 39.69 | 47.79 |
| ZEB2 | 2657.37 | 3409.74 |
| DDIT4 | 31.02 | 106.66 |
| PIM1 | 39.83 | 44.51 |
| BNIP3 | 5.96 | 4.72 |
| SLC2A1 | 4.68 | 6.36 |
| LDHA | 120.40 | 322.04 |
| 1 PGK | 227.89 | 393.22 |
| HK2 | 45.71 | 24.61 |
| PFKFB3 | 112.53 | 171.48 |
| SLC16A1 | 17.87 | 16.41 |
| GAPDH | 708.89 | 992.79 |
| ENO1 | 285.22 | 546.44 |
| PKM | 205.71 | 301.94 |
| MYC | 10.13 | 17.44 |
| PDK1 | 32.30 | 26.26 |
| SLC16A2 | 0.11 | 0.00 |
| SLC16A3 | 76.69 | 139.69 |
| CPT1A | 97.27 | 117.53 |
| GLS | 181.21 | 225.22 |
| ACLY | 28.45 | 29.74 |
| CSF1R | 74.72 | 82.25 |
| IL12A | 0.14 | 0.41 |
| TNF | 13.46 | 10.26 |
| CSF3R | 311.18 | 347.89 |
| IL18 | 18.58 | 23.79 |
| IL6 | 0.38 | 0.00 |
| CSF2RB | 24.55 | 29.54 |
| CSF2RA | 139.44 | 160.82 |
| CSF1 | 1.13 | 1.64 |
| IL1B | 149.86 | 33.43 |
| IFNG | 4.20 | 3.28 |
| APOE | 0.14 | 0.00 |
| APOC1 | 0.19 | 0.21 |
| APOC2 | 0.11 | 0.00 |
| SORL1 | 262.12 | 201.22 |
| ABCA7 | 19.60 | 14.36 |
| PSEN1 | 248.90 | 281.63 |
| PSEN2 | 2.64 | 5.95 |
| APP | 123.05 | 119.18 |
| TMEM106B | 19.62 | 19.08 |
| GRN | 310.05 | 488.60 |
| ABCC9 | 0.17 | 2.46 |
| KCNMB2 | 0.26 | 0.62 |
| APOA1 | 0.20 | 0.00 |
| APOC1.1 | 0.19 | 0.21 |
| APOC2.1 | 0.11 | 0.00 |
| LDLR | 78.68 | 97.02 |
| ABCA1 | 90.40 | 70.77 |
| CETP | 2.23 | 1.64 |
| HMGCR | 26.28 | 45.33 |
| SRARB1 | 0.00 | 0.00 |
| PLTP | 0.76 | 0.62 |
| APOA5 | 0.00 | 0.00 |
| CYP27A1 | 61.19 | 67.07 |
| SORT1 | 82.73 | 51.69 |
